# A Digital Tool to Build the Capacity of Leaders to Improve Working Conditions Related to Psychological Health and Well-Being in Teams: Intervention Approach, Prototype, and Evaluation Design of the Web-Application “wecoach”

**DOI:** 10.3389/fpubh.2020.521355

**Published:** 2020-12-18

**Authors:** Luisa A. Grimm, Georg F. Bauer, Gregor J. Jenny

**Affiliations:** Center of Salutogenesis, University of Zurich, Epidemiology, Biostatistics and Prevention Institute, Zurich, Switzerland

**Keywords:** digital intervention, evaluation, workplace health promotion, occupational health psychology, leadership, capacity building, team development

## Abstract

The “wecoach” is a web-application that builds the capacities of team leaders to improve working conditions that are positively related to the psychological health and well-being of their team members. The web-application works through an automated, rule-based chat enhanced by machine learning. This so-called conversational agent guides the team leader through a systematic project cycle, providing a mind map of work and health, training materials, self-assessments, and online tools to conduct team surveys and workshops, as well as self-evaluation of progress and effectiveness. In this paper, we present the development process of this web-application, which resulted in (1) a comprehensive intervention approach, (2) the prototype, and (3) the implementation of an evaluation design for a multi-level, randomized controlled trial.

## Introduction

The aim of this paper is to present a web-application that builds the capacities of team leaders to create working conditions that are positively related to the psychological health and well-being of their team members. To achieve better health and well-being of employees, researchers in the field of occupational health psychology (OHP) have been developing novel approaches in regard to the level and design of interventions: First, the level of interventions has seen shifts from bottom-up individual health behavior and top-down health management strategies to the middle level of leaders and teams, who are empowered to continuously improve their working conditions (1, 2). Evaluation research of organizational-level interventions has shown that line managers play an important role for successful implementation of such interventions (3–5). As a consequence, it has been proposed to build capacities of leaders and of their teams to sustainably optimize their working conditions (1, 6, 7). Second, digital intervention designs have found their way into OHP. Digital interventions are already popular in therapeutic contexts and meta-reviews of their effectiveness have been published as early as 2012 (8). In the field of OHP, digital interventions support individuals with issues such as stress, anxiety or depression at work, and systematic reviews show that they tend to be effective (9–12). Similarly, the field of human resources management increasingly uses e-solutions for personnel selection and development—including leadership coaching and training, or apps for promoting employee engagement. The web-application presented in this paper combines both a digital and a team-level approach. It builds the capacities of team leaders to assess and improve the working conditions of their team through a participatory team development process. It works through an automated, rule-based chat; this so-called conversational agent guides the team leader through a systematic project cycle, providing a mind map of work and health, training materials and self-assessments. Further, it provides online tools to conduct team surveys and workshops, as well as self-evaluation of progress and effectiveness. Additionally, a machine learning component has been implemented to personalize the rule-based chat, based on growing context, process and outcome data generated by the users. In this paper, we first present the background and development process of this web-application, which then resulted in (1) a comprehensive intervention approach, (2) a prototype labeled “wecoach,” and (3) the implementation of an evaluation design for a multi-level, randomized controlled trial. At this point, empirical results of the evaluation are not yet available.

## Methods and Materials

### Background of the Intervention Approach: Capacity Building

Based on previous research in occupational health psychology (OHP), including our own intervention and evaluation studies, we established *capacity building* as the guiding principle underlying the development of our intervention approach (1, 6, 7, 13, 14). Capacity building has its roots in the fields of community development and foreign aid (15, 16) and was identified as a way of increasing and sustaining the effectiveness of health promotion programs (17). It has become a highly recognized approach and is referred to as “(…) the process of enhancing the ability of an individual, organization or a community to address their health issues and concerns.” (p. 59) (18). Similarly, the WHO's “International Classification of Health Interventions” (ICHI) lists “capacity building interventions targeting behaviors related to psychological health and wellbeing”: These are defined as “providing resources or initiating strategies to increase the ability of an organization (...) to address health issues by creating new structures, approaches or values in relation to patterns of behavior that may affect psychological health and well-being.” (VEL.VA.ZZ; see https://mitel.dimi.uniud.it/ichi/). Capacity building has been applied across various contexts and thus is a rather broad and flexible approach, yet there are a few key principles to it, which we characterize as following in relation to workplace health promotion (13): First, it is described as a *multi-level approach* that comprises micro-, meso-, and macro-levels, for example, the individual employee and leader, the team, and the entire company. Second, it refers largely to a *systemic approach* that underlines the importance of connecting to the self-referential logic of social systems. In this view, interventions aim at influencing self-organization, -monitoring and -optimization of social systems through bringing the system's stakeholders into communication, exchanging multiple (maybe contradictory) perspectives, producing visibility and a common language, and finally triggering mutual learning and action toward better work and health (19). Third, it is an *enabling approach* that aims—as a matter of principle—at independence from external support in the long run (13). Of course, this doesn't exclude the option of utilizing supporting services by business consultants or coaches, but not in terms of delegating actions (and responsibilities) that should be internally appropriated. Fourth, it is a *developmental approach* and therefore both process (“building”) and outcome (“capacities”) (13, 19): Individual and organizational capacities are identified and strengthened, forming the ground for healthy working processes (20) and continued health-oriented self-optimization. Fifth, it is a generic concept and therefore pursues a *contextual approach*, that is, interacting with the system's structure, strategy and culture, but also the competencies, motivation and identity of the members (13, 19). Starting with these general guiding principles, the detailed *intervention theory* was developed, from which the overarching *intervention architecture* (i.e., the project cycle) and the specific *intervention elements* (such as team surveys) were derived. Essentially, the envisaged intervention architecture had to contain two phases of capacity building: A phase one, where the leader is informed and trained on her/his own (“team leader training”), and a phase two, where she/he engages with the team to improve their working conditions (“team development”).

### Rapid Prototyping of the Web-Application

**Figure 1:**
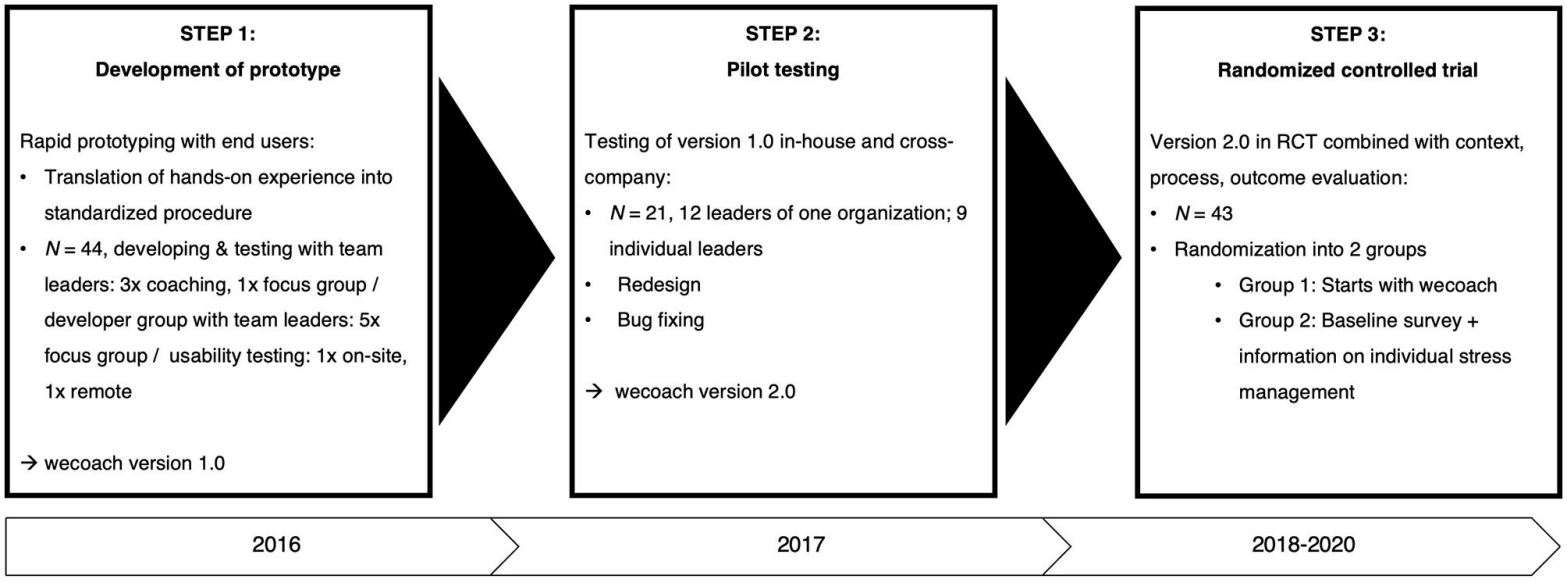
Development process. Prototype development, pilot testing, and RCT.

Based on the intervention approach—that is, the intervention theory, architecture and elements—the prototype development was initiated. Here too, in line with capacity building, our assumption is that developing solutions for health promotion in organizations has to be done together with the people, rather than for people (21). Following rapid prototyping approaches in public health (22), we developed the prototype in three steps (Figure 1), whereby the last step—the randomized controlled trial—is presented separately (see below).

First, a prototype was developed together with the end users: 44 leaders were recruited who were willing to participate in coaching and focus groups which took place every 2 to 3 months. One group of leaders was recruited from a health care organization; they played through the designed intervention step-by-step, coached by the developers in three meetings and then reflecting on the process in a focus group. Another group of leaders was recruited from a range of heterogenous, small to large organizations. They took part in five consecutive focus groups with the developers; in the forefront of each focus group, they were delivered specific tasks and questions to ensure that their requirements were considered in every step of the development of the wecoach. These tasks were focused on key parts of the intervention architecture and elements, such as the training of the team leader, the team survey or the team workshop. Second, this prototype version 1.0 then underwent a pilot testing phase in organizations as well as with individual leaders. A heterogeneous group of 21 users tested the wecoach and were then questioned on users' satisfaction and acceptance (23). Third, with these results, the prototype version 2.0 was released with a redesign and bug fixings. This version was the basis for designing a multi-level, randomized controlled trial (RCT).

### Designing a Multi-Level, Randomized Controlled Trial

The evaluation design had to mirror the prototype's two-phase capacity building approach, that is, the “team leader training” (phase 1) and the “team development” (phase 2). The main research question regarding the *outcome of phase 1* was if the training of team leaders will increase their feeling of self-efficacy to conduct the team development. The main research question regarding the *outcome of phase 2* was if the working conditions will improve through the team development—in terms of lower job demands and higher job resources, which are known to be related to the psychological health and well-being of employees (24–26). In both phases, not only outcome variables must be taken into account, but also *process and context* factors. The wecoach was designed to unobtrusively generate subjective and objective data on the intervention's context, process and outcome, in reference to the CPO model for evaluating organizational health interventions (27). The question regarding the *process of phase 1 (‘team leader training’)* was if leaders who closely adhere to the process and thus objectively exhibit longer duration of use will experience a pronounced increase of self-efficacy (28); this would also be expected from leaders who reveal a favorable system appraisal as a subjective process factor (23). In regard to the *context of phase 1*, we asked the question if a leader's health awareness and her/his leadership style will positively influence the above outlined process factors; that is, a leader who is aware of the factors that stress and engage her/his team at work, and who has a routine of actively leading change as a committed role model, involving and appreciating the team, will also engage her-/himself more actively within the wecoach (29). The question regarding the *process of phase 2 (“team development”)* was if higher participation rates of the team members as objective process factor will amplify positive changes in job demands and resources; this would also be expected from favorable appraisals of the team development as subjective process factor (30, 31). Context factors of team development processes are often discussed in OHP research and mostly examined through qualitative data (27). Through the wecoach, we also collect quantitative context data, such as team climate, that potentially are of relevance to the change process (32). Most importantly, in regard to the *context of phase* 2, we asked the question if successful capacity building of the leader—measured as self-efficacy—acts as a moderator of the team development process, which constitutes the basic premise of the wecoach: Empowering a team leader for team development has to manifest itself in a participatory team development that is positively appraised by the team and thus leading to positive changes in their job demands and resources. Finally, evaluation of change in teams must respect the nested data structure and the dynamics of change in the respective unit of change. For this reason, analyzing the effects of an intervention in teams should apply corresponding approaches to the study design: This involves randomizing teams and not individuals into an intervention and control group, and control for cluster affiliation in data analysis. Whereas in organizational level interventions within-group variation can be considerable, teams constitute a more distinct unit of change, to which the intervention design is aligned to. Thus, randomizing teams may help eliminating the effect of confounding broader contextual factors (33).

## Results

### Resulting Intervention Approach

**Figure 2:**
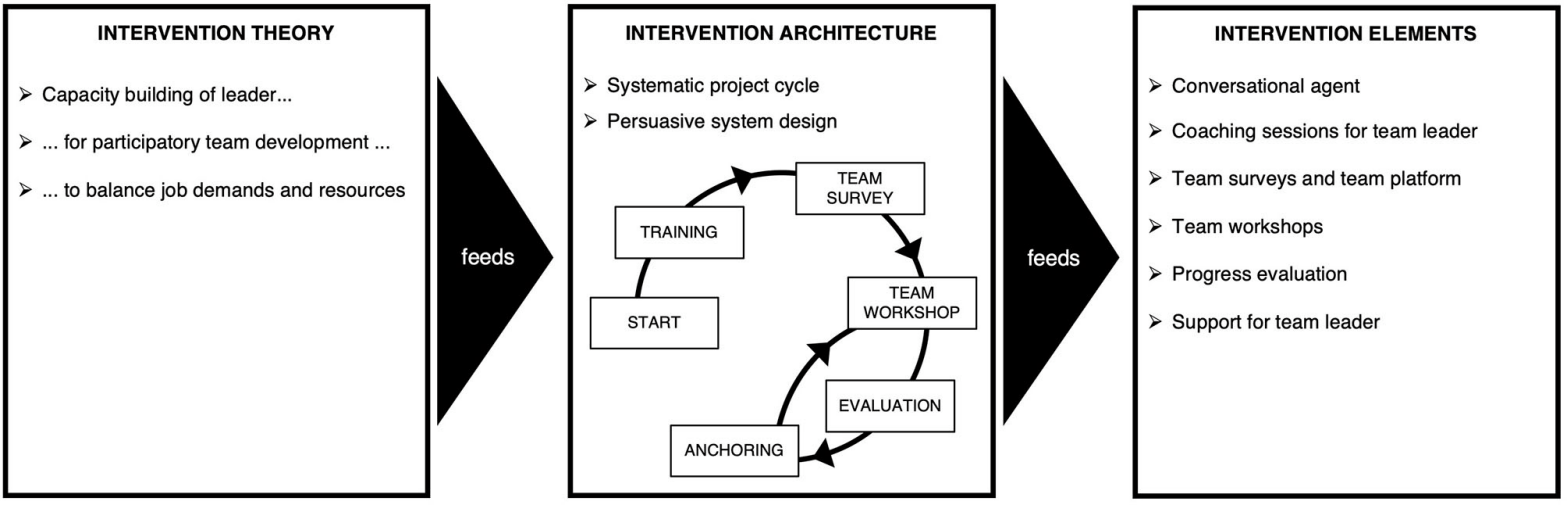
Overview of the intervention approach. Theory, architecture and elements.

The resulting intervention approach underlying the wecoach is divided into three sections: The intervention *theory* that feeds into the intervention *architecture*, which feeds into in a range of intervention *elements* (Figure 2). These are described in the following paragraphs in detail.

#### Intervention Theory

##### Capacity Building of Leaders for Participatory Team Development

We characterized capacity building as a *multi-level, systemic, enabling, developmental*, and *contextual* approach. This general guiding principle underlies the following detailed approach to enabling team leaders to conduct a participatory, contextualized, and self-guided team development (14). We target *teams* (and not organizations), as these are distinct, smaller units of change that can achieve results more quickly through direct involvement of team members and short decision chains, which not only facilitates communication on change but makes effects directly visible and noticeable in day-to-day work (2, 34). Improvements can also be better tailored to the specific team context, and local team-level solutions are more feasible to implement compared to macro-level, organization-wide changes (35). As a consequence, team level workshops have proven to be an essential ingredient of organizational-level interventions (1, 2). The team level may also intensify psychosocial mechanisms of change, such as fostering mutual learning, building social identity (i.e., setting mutual goals, addressing underlying values, and reflecting shared mental models on work and health), creating shared meaning through the intervention's topics and targets, or triggering (positive) emotional contagion during participatory action (35). *Leadership* research has seen an increasing interest in the impact of leadership on employee health, and meta-analyses report moderate correlations between leadership and health of employees (36–38). The causal mechanism between leadership and health have also been discussed (39–41): Leaders influence, for example, how work is organized, they define the contents and requirements of work, they shape the working conditions and the relationships at work, all of which are known to potentially impair workers' health—or foster their engagement and well-being. Another line of research has explored how leaders' health awareness and behavior is related to the members' health behavior and health status (29, 42–44). Leaders also play a pivotal role in change processes (3–5): Their commitment, support, and transparency is vital to the success of interventions. *Combining team and leadership* research with capacity building as outlined above, team leaders are advised to adopt a participatory approach, which is strongly recommended by intervention research (45, 46), and conduct the change process *together* with their team. Literature shows that participation influences the intervention outcome positively in several aspects: Aust, Rugulies, Finken and Jensen (47) showed in their study that employees react negatively to only having a limited influence on an intervention. In line with that, Lines (48) reported that the amount of participation of employees was positively related to commitment to the organization as well as goal achievement, and negatively related to resistance of change. Furthermore, Nielsen, Randall and Albertsen (49) examined in a study whether participation in change is associated with stress and job satisfaction and could show that high levels of participation correlated with low levels of behavioral stress symptoms, as well as higher post intervention job satisfaction [see also (50)]. Participatory approaches may also increase the perceived fit between the intervention's design and both the team and the leader's attitudes, appraisals and actions (3).

##### Balance Job Demands and Resources

Leaders and teams will require a mind map on work and health, to facilitate exchange and action on optimizing working conditions and to provide mutual targets of change. Such a mind map may develop into a capacity itself, that is, a shared mental model and common language on work and health in the team (14). In this regard, the job demands-resources model (JD-R) (51) has been identified to be best suited for this purpose (7). In research, ample evidence supports its heuristic relevance for explaining the interrelations between work and health (51, 52). In practice, it promotes not only a negative, but also a positive view on work and health, which is ideal for storytelling and generating energy for action. Two processes are of importance in this model: On the one hand, the motivational process, describing how job resources (e.g., social support, role clarity and autonomy) lead to high work engagement, low stress levels and high performance. Such resources-rich workplaces foster the willingness of employees to invest their skills into a work task. On the other hand, the health impairing process describes how chronic workload depletes the mental and physical abilities of employees. This leads to stress and exhaustion, and health problems are the result. Thus, interventions are recommended to address not only the reduction of job demands, but also the equally important strengthening of job resources. In this line of argument, key job resources such as role clarity, decision latitude or social support are created and stabilized collectively on the team level (24)—and thus can be best improved on this level as well. Further, previous research has consistently shown the relevance of job resources for both work engagement and burnout (24–26), which has led to an increased focus on both of these outcomes to ensure a holistic intervention approach (14). Thus, a mind-map based on the JD-R model may serve as a central element in building a narrative of balancing job demands and resources to achieve better health and well-being (53): This storytelling of “work and health” as a two-fold matter (demands-resources, stress-engagement) is designed to be as comprehensible and meaningful as possible to both team leaders and team members so that it becomes part of the team's discourse and culture—a capacity, as noted above. This requires easy-to-grasp visuals, keywords and story elements that can be applied throughout the training, the display of survey results and the development of measures in workshops, for example. This can be described as a process where existing beliefs and narratives on work and health are transformed into a “new truth” and are validated collectively (54).

#### Intervention Architecture

##### Systematic Project Cycle

Most OHP interventions follow an intervention cycle with several steps, comprising stages of preparation, action, and anchoring (27, 55, 56). We designed the intervention architecture as a project cycle consisting of six steps, with each step having one to five coaching sessions to be worked through by the team leader online (total of 20 sessions). Step 1 and 2 comprise the first phase of team leader training, while step 3 to 6 comprise the second phase of team development. In the first phase, the team leader is provided with a mind map of work and health, learning materials and tools to conduct team surveys and workshops. This is supported by short assessments triggering self-reflection regarding the context of the team development, and helping her/him to identify facilitators and overcome barriers on individual, team, and organizational level. The coaching sessions are designed to build up capacity and awareness for work and health step-by-step, and strengthening the leader's skills, readiness and self-efficacy for the team development. In the second phase, the team gets involved. The team leader will prepare and conduct a team-survey as learned in steps 1 and 2. Based on the results of the survey, she/he receives support for the planning and implementation of a workshop, as well as the evaluation of the team development process and effect. A short session concludes the project cycle with recommendations on how to incorporate this process into daily routines.

##### Persuasive System Design

This intervention architecture builds not only on OHP intervention and evaluation research—which integrates the broader field of organizational change and management research—but also on the field of (persuasive) system design (57–59): Task and dialogue support are provided, as leaders are guided—but not forced—by a conversational agent through the series of coaching sessions (so-called “tunneling”). Each coaching session is devoted to one task (e.g., training on how to conduct a team survey) and contains visually attractive online learning materials, some of which can be downloaded. Guidance is tailored to contextual factors and personalized according to the user's demands (e.g., offering and requesting in-depth information). As the leader is recommended to follow a sequence of coaching sessions, tailoring is currently limited to variation *within* the sessions, but not between sessions. Suggestions vary depending on team size, readiness for change, or the results of the team survey, for example, which can be constantly refined and tested (see below too, machine learning). At the end of each coaching session, the user is praised for completing the session and asked if she/he would like further suggestions on where to continue in the process. The team leader will first rehearse the team development in the training sessions, before informing the team and “going live”; that is, she/he will conduct a survey on working conditions for her-/himself and develop exemplary measures utilizing the methods that will be applied later with the team too. This whole process includes various tools for self-monitoring and self-reflection, where the leader will self-assess her/his self-efficacy, health awareness, or leadership style during change, latter of which is also assessed by the team and presented as perceptual distance to the leader. Further tools for process and outcome monitoring including the team survey are provided to stimulate intermittent self-observation. Identified as important design features (57, 58) are automatic reminders and exchange options between leaders using the wecoach. This way, social support, mutual learning, cooperation and facilitation could be fostered, but also social norms influenced, where leaders compare themselves—maybe even compete—and recognize their achievements. This outlined intervention architecture feeds into a range of intervention elements.

#### Intervention Elements

##### Digital Coaching Through a Conversational Agent

The chatbot, referred to as the *conversational agent* in this paper, is considered to be the main intervention element, because it guides a team leader closely through the whole project cycle. A conversational agent is a real time chat function, that is, a text based dialogue system with one area each for text input and text output, which enables a person to interact and communicate with a technical system (60, 61). Conversational agents are designed to interact with an individual in a way that imitates human interaction and makes individuals apply typical social interaction behaviors to the human-interface-setting (61). They offer a novel approach to team development by complementing, extending or even replacing face-to-face coaching (61). The application of conversational agents with a health focus is novel and therefore little is known about what individuals need exactly when interacting with them (62). This is also due to the fact that an appropriate design of a conversational agent requires knowledge of various disciplines such as human-computer interaction, computational and sociolinguistics, psychotherapy, and motivational interviewing (60). The conversational agent designed for the wecoach covers a range of coaching techniques (e.g., questioning, clarifying, scaling of perceptions, goal setting etc.) to help leaders shift their perspectives and thereby discover different approaches to achieve their goals (63). As coaching supports individuals in developing their own, tailored solutions, it supports the capacity building of leaders as well as the adaptivity of the intervention to diverse organizational and individual contexts, a factor that enhances their adoption and effectiveness (64).

##### Coaching Sessions

During the *coaching sessions* the conversational agent provides the team leader with online materials, tools for self-assessments as well as for the team survey and team workshop, as described above. Through closed questions, for example, the agent assesses emotional reactions at critical moments (such as results of surveys) and provides input on how to deal with them. Similarly, critical situations that may arise (such as conflicts in workshops) are pre-emptively addressed and learning materials presented. The sessions' contents are derived from OHP intervention and evaluation research as delineated in the intervention theory (14), but also includes success factors from organizational change literature (2): For example, the team leader is encouraged step-by-step to communicate promptly about the process, create a shared awareness and purpose of change, define goals and develop a mutual vision of the future work situation, produce quick-wins in the participatory workshops, and monitor as well as feedback progress to the team.

##### Team Survey and Team Platform

A central intervention element is the *team survey*, that can be accessed through a *team platform* (see below, user interface). Through a series of sessions, the team leader prepares and activates an online team survey to assess the current level of stress and engagement at work, job demands and resources, and exhaustion and psychological well-being in her/his team, which is based on the mental model of work and health provided as a common thread throughout various sessions. Job demands and resources, for example, are assessed with validated scales from the HSE Stress Management Standards that have proven to be widely applicable in diverse branches of business and being relevant for well-being, health and productivity (65). Additional scales can be complemented to cover specific needs of quantified assessment, such as interprofessional collaboration in the health care sector.

##### Team Workshops, Progress Evaluation and Support

Based on the automatically generated results from the team survey, the team collectively develops actions to improve their balance of job demands and resources in a *team workshop*. The workshop is moderated by the team leader, who has been instructed by the conversational agent and provided with guidance material to organize and conduct a team workshop. Built-in *process evaluation* provides feedback from the team to the leader by means of two quick surveys of both perceived progress and impact of the team development. The conversational agent also helps the leader through a series of questions to identify her/his need of *support* and refers to corresponding coaches who are adept with the wecoach. Hereby the option will be considered that team leaders and teams can exchange their experience with other team leaders and teams on a voluntary basis—ideally matched by an algorithm based on contextual and procedural data gathered during the use of the wecoach.

### Resulting Prototype

#### User Interface

**Figure 3:**
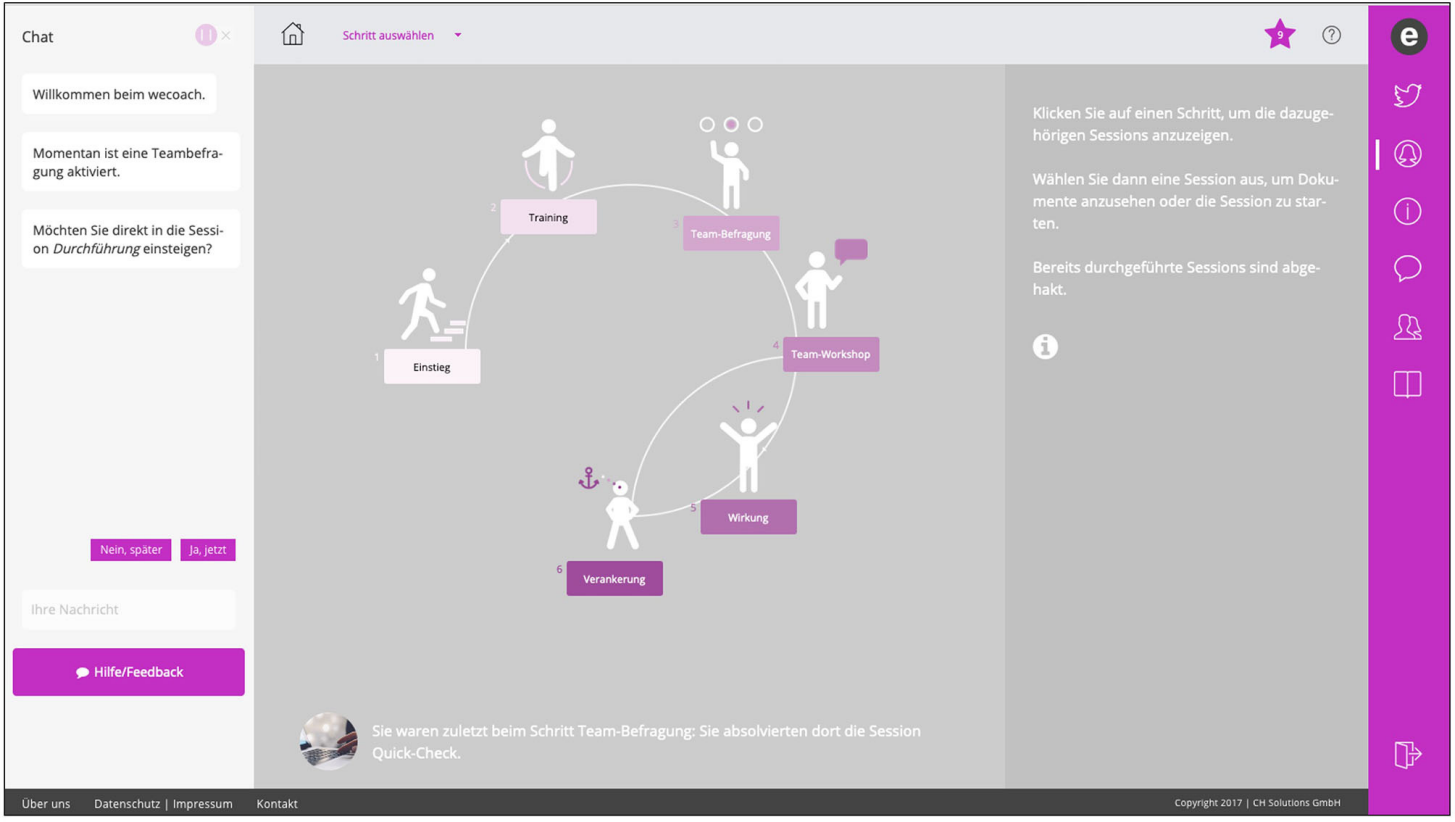
Screenshot of the wecoach prototype. Version 2.0, German only, depicting the project cycle with steps 1 to 6.

As a result of the focus groups, the wecoach was developed as a web-application and not as a native application or software program. Team leaders expressed that installing new software in their companies faces many unnerving obstacles, which would inhibit the implementation of the wecoach. Thus, the prototype was developed for application in browsers, such as Google Chrome or Firefox, using extensible markup language (XML) and HTML 5 for rendering the conversational agent and the coaching contents (https://wecoach.ch). The final user interface was designed to provide a maximum of space for displaying the contents (such as learning materials or self-reflective questionnaires). The conversational agent is placed in the chat window left of this so-called “workspace” (Figure 3): It interacts with the user after login through messages, and advises the team leader in a main dialogue on the next action to take and what coaching session to complete, as described above. It asks mainly closed, but also some open questions to be answered by the user. The current version of the wecoach contains ~2,500 chat messages that are triggered in a main dialogue and the specific coaching sessions. Further, the wecoach contains ~100 online materials that are displayed in the workspace during the coaching sessions.

Two additional interfaces were built for the team development in phase 2: To fill-out team surveys, the team members can log into a team platform and add the team they belong to (https://wecoach.me). This platform will act in future as a linkage between individual team survey results and a planned app that generates data on personal strategies to improve the working situation and balance work-life demands (“mecoach”). Importantly, these data are only visible to the respective individual data owner. The team workshop can be conducted via a separate website, where team members can brainstorm and develop actions simultaneously through digital worksheets (based on Etherpad, https://etherpad.org). Alternatively, they can work with printed worksheets provided by the wecoach as PDFs or with Word templates to be saved on their laptops.

#### Software Architecture and Machine Learning

**Figure 4:**
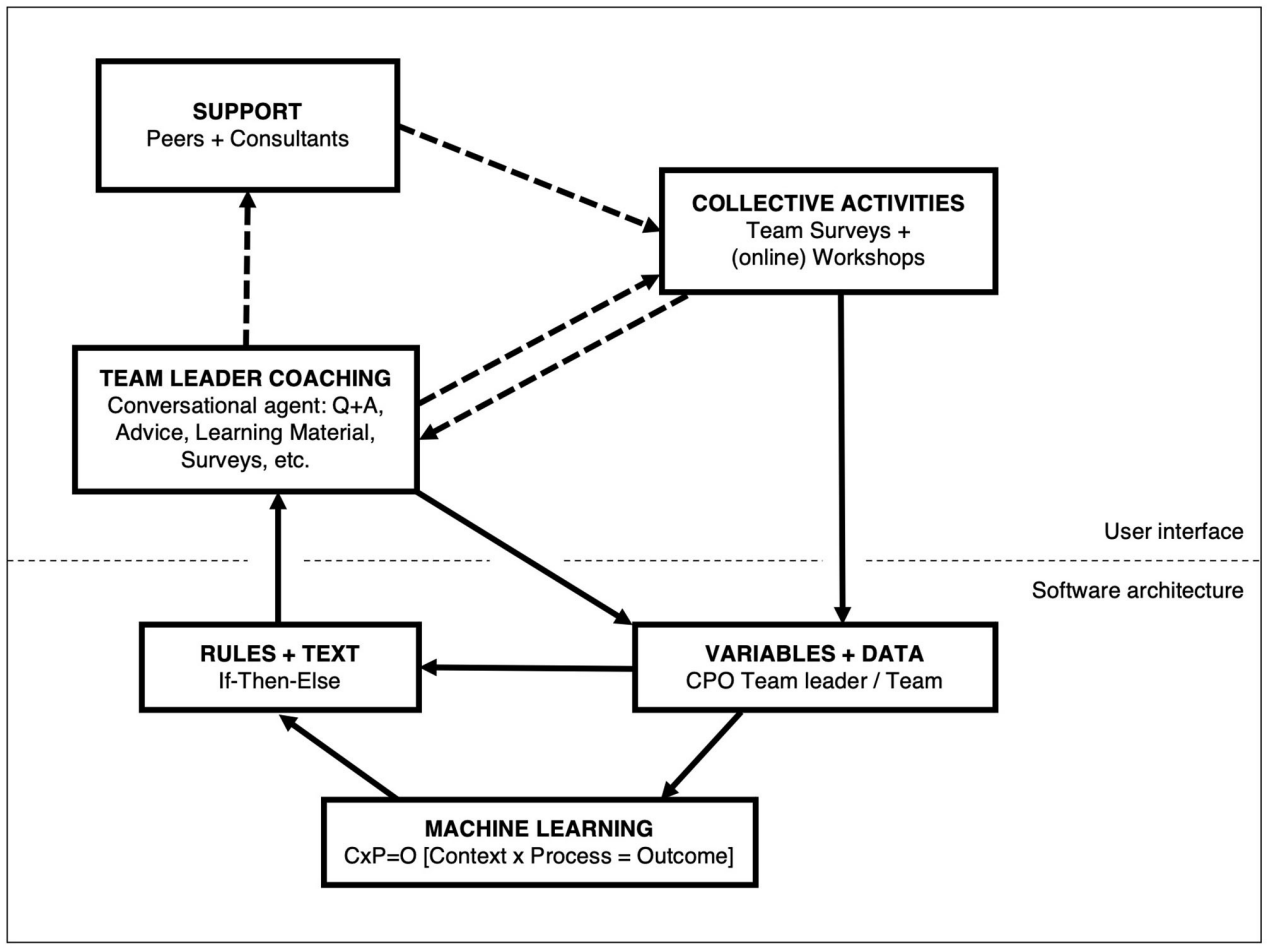
Overview of the software architecture. Data driven capacity building.

The software architecture constitutes a data-fueled “motor” which feeds and is fed by the team leader training and the team development (Figure 4): During the coaching sessions and through the team surveys and workshop, data is inherently collected regarding a broad range of variables (see evaluation design too). These variables cover the context, process and outcome (CPO) of the entire process (27): For example, contextual factors such as team size, team readiness and team climate, perceived hindrances and challenges for change, leadership style, self-efficacy, and health awareness are collected quantitatively and qualitatively as part of leader self-reflection and as preparation for the team development. Similarly, process factors such as leader compliance are assessed, through registering if forms on various exercises have been filled out (without semantic analysis), or collecting appraisals of the team development by quick surveys presented to the team, who rate workshop quality, output satisfaction, and outcome expectancies (32), as well as intermittent “impact assessments” of changes in the working conditions (2). Additionally, during interaction of the team leader with the conversational agent, responses to questions are registered, for example, if further information on a topic like team climate is desired. Latter interaction alone produces a set of nearly 300 data points, which is complemented with dates of login, session start and end dates, session frequency counters, and more. Outcome data is mainly collected through the team survey initiated by the team leader, covering the mental model of work and health provided by the wecoach.

This data feeds the conversational agent through “if-then-else-rules.” Based on the users' answers, the rules trigger the next text message. These messages will eventually lead to collective activities of the leader together with her/his the team, that is, conducting team surveys and workshops. These activities again generate data that are transferred back into the database. If a leader requests support through an external consultant (or peers, in future) suggestions will be made and corresponding actions registered to the database, as far as they are visible to the system (i.e., through questioning the team leader). In essence, the wecoach aims at providing a digital answer to the question “what works for whom in which circumstances?” (66). A first version of a machine learning component has been implemented, which capitalizes on the CPO database outlined above. With increasing numbers of users completing a project cycle, it will register what process factors relate to change in outcomes, and cluster the contextual attributes of these successful processes. This information will allow to adapt the “if-then-else” rules—in a first step manually, in future potentially automatically.

#### User Experience

The team leaders in diverse organizational contexts and with a range of leadership experience perceived the idea behind the wecoach as worthy of engagement and the resulting prototype as being fit for its purpose, and viewed as being not too academic and not too trivial either. At the same time, a majority of those potential users reported a heavy workload and lack of time, and therefore the worry of not being able to integrate the wecoach into their daily business. Other team leaders remarked that their “team” lacks cohesion and interdependency, or includes further hierarchies, all of which may complicate a “team development.” Furthermore, we experienced a high need of support in all stages of the process, which means that users need to be very well-guided. Another factor in this regard is the self-navigation through such an online tool, especially when users do not follow the suggested course that is structured in a comprehensive, systematic project cycle. It was expressed that a “fast track” option would be desirable, without compromising the idea of capacity building. Finally, as often observed when surveys are applied to create visibility through numbers, much discussion arises on content and methods (53, 67): All team leaders viewed the team survey as a critical moment and wanted to make sure that they could absolut correctly interpret and discuss the results with their teams.

### Implementation of the Evaluation Design

#### Data Collection for Context, Process and Outcome (CPO) Evaluation

**Figure 5:**
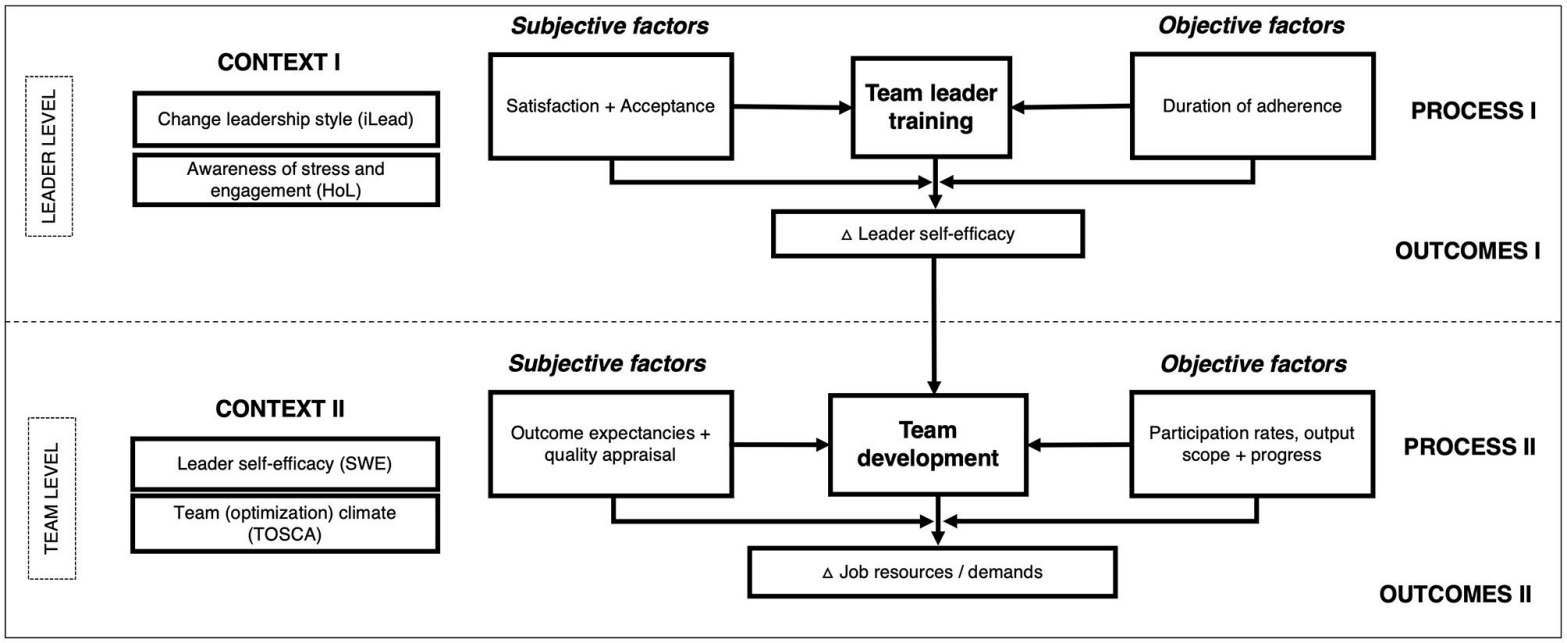
Overview of the evaluation design. Data collection.

From the breadth of data collected within the wecoach, a selection had to be made for the randomized controlled trial of the prototype. Based on the research questions formulated in the methods section, complexity was reduced to two main outcome indicators and a selection of process and context indicators (Figure 5), clustered into the two phases of capacity building.

##### Data for CPO Evaluation of the Team Leader Training

As *outcome indicator* serves a self-efficacy scale developed for this specific purpose, based on Bandura's template for self-efficacy scales (68). The scale captures self-rated facets such as being confident explaining how health, stress and engagement are influenced by working conditions, conducting a team survey and feedbacking results, developing measures in a workshop, and mediating between differing opinions. A scale by Wixom and Todd (23) has been applied as *subjective process indicator*, rating the team leader's system appraisal (i.e., satisfaction and acceptance). System use (i.e., duration of adherence) as an *objective process indicator* will be calculated from the automatically registered session start and end dates. This will be matched with data on the trace of clicks during a session, that is, the minimally to maximally possible amount of clicks on response buttons during a session. Further indicators of adherence will be explored, such as patterns of fidelity (i.e., a linear or volatile way of proceeding through the wecoach) or concentration (i.e., usage of the wecoach during few cohesive periods or spread over time). As *context indicator* an adapted version of the Health-oriented Leadership scale has been applied (29), assessing a team leader's awareness for stress and engagement in her/his team, as well as a modified version of the iLead scale (69) that measures a team leader's behavior style during change processes.

##### Data for CPO Evaluation of the Team Development

As described earlier in regard to the team survey, the Management Standards Indicator Tool established by the British Health and Safety Executive (HSE) has been applied (65) to compute the *outcome indicator* of job demands and resources. It consists of six subscales capturing quantitative *demands, control, support* from supervisors and colleagues, negative *relationships, role* clarity, and transparency during *change*. These subscales have been complemented by three adapted scales of the SALSA questionnaire (70), assessing job *variety*, competency *development* and *overextension* (i.e., a qualitative overload in the sense that work is too difficult in relation to one's skills). As *objective process indicator*, participation rates will be mainly assessed through response rates to the various team surveys. Additionally, participation rates at workshops are assessed by the team leader within the wecoach by means of a form. As *subjective process indicator*, a quick survey can be initiated after the team workshop to assess appraisal of the workshop quality, output satisfaction, and outcome expectancies (32) —as perceived by team. Team appraisal is also contrasted with the leader's perspective, as perceptual distances have shown to be of relevance in change processes (3). As *context indicator*, the “Team Optimization Climate Scale” (TOSCA) has been developed for this purposes: A team that experiences high inclusion and respect of diverse opinions, that values its strengths and is solution-orientated, and aware of their well-being and health—as derived in the intervention theory (14) —may participate and engage themselves in the process and thus also appraise it positively.

#### Recruitment of Participants and Randomization

**Figure 6:**
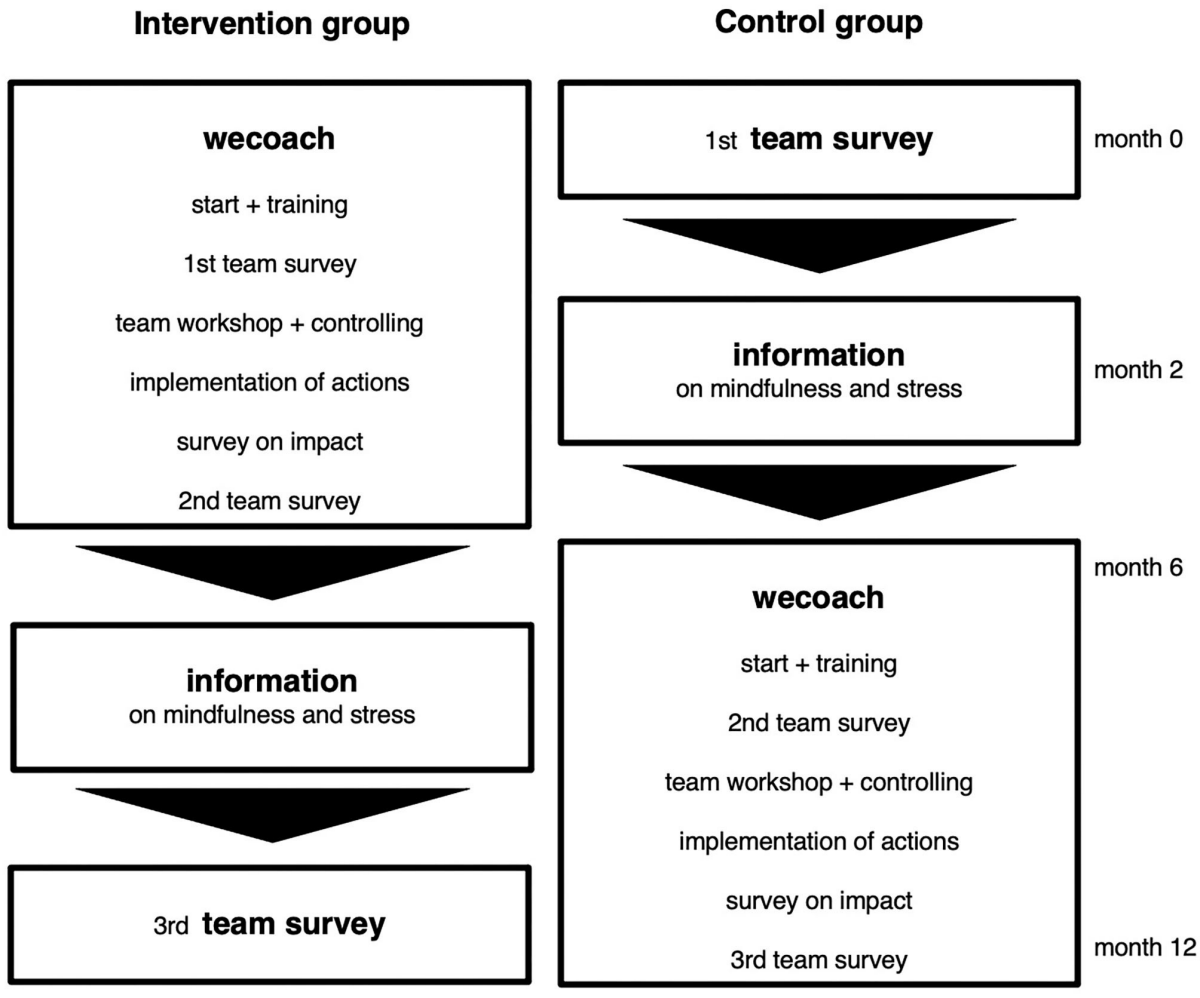
Study design. Randomized waitlist control group.

Study recruitment was done through on- and offline marketing such as posting on social media (e.g., LinkedIn), sending newsletters through university channels and professional networks, organizing information events, advertising during talks and appearances, and directly contacting company representatives. During the recruitment process, team leaders confirming their study participation were randomized in order of registration into an intervention and control group. One group started directly with the wecoach while the other completed a baseline survey and while waiting were provided with information on individual stress management (Figure 6). The recruitment process lasted from May 2018 to August 2019, yielding an initial sample of 22 teams in the intervention group and 21 teams in the control group.

During the recruitment phase of the *randomized controlled trial*, we had difficulties reaching a sufficiently large group of participants, as can be seen from the extended time spent for this phase and the final number of participants. Due to the novelty of the approach and the interplay of multiple components that naturally come with such a digital solution to team development, quickly grasping what exactly the wecoach is about, and what the logic behind it is, proved to be a challenge for most users. Since such a digital solution is a new approach to team leader training and team development processes, it posed a communication challenge to explain what an online program like the wecoach can do, and can't do.

## Discussion

The development of the wecoach—from theory to prototype to evaluation—has been an endeavor starting 4 years ago and is still continuing. The decision to digitalize a capacity building process involving team leaders and their teams added systemic complexity to an already complicated process, that is, applying digital technology to change health-related behavior. Based on the results of the prototype development and implementation of the randomized control trial, we can conclude the following: A novel intervention approach as the wecoach needs to be accompanied by a clear strategy showing potential users (organizations and individual leaders) the added value of adopting such a tool. Once adopted, the system has to guide users quickly and efficiently through the process, to get and keep team leaders with high workloads on board, without compromising the underlying approach of capacity building (limiting “fast track” options to a certain extent). If this can be accomplished, an application like the wecoach can promise an accessible, efficient and also highly scalable solution for improving working conditions in teams, fostering visibility of and continuous communication on the topic. It is a limitation of this paper that evaluation results are not yet available to provide further insights into the context, process and outcome of this approach. In this regard, due to data collection being inherent to the coaching process, the wecoach has the potential to collect large—and cheap—data for context, process and outcome evaluation of an OHP intervention, with high external validity. However, the wecoach is currently limited to subjective data. It could be beneficial to examine if its application is not only related to changes in job demands and resources, as presented in the evaluation design (Figure 5), but also to objective measures of health. Hereby, different stakeholder perspectives might be taken into account by the evaluators. For example, from a research perspective, linking changes in subjectively perceived working conditions to sick leave data in a team would further strengthen the evidence-base of workplace health interventions. From a business company's view, reduction in a team's health-related absenteeism rates could make a strong case for adopting and implementing such novel solutions. In future, health-related data generated through passive smartphone sensing methods might also be taken into account, which could be achieved through linking the wecoach web-application with the envisaged mecoach application for smartphones—provided this field of research yields reliable, valid and also applicable methods. In any case, team development is a high-energy effort: A digital approach may lower thresholds on the long term, but may first bring along an increased cognitive load on behalf of the team leader, who has to engage in the training, trigger surveys and moderate workshops. Although this appropriation enhances the odds of effectiveness—as seen in “live” studies (2) —it is easier to delegate such processes to consultants and coaches. Such delegation not only reduces investment of energy, but also responsibility for the actions and outcomes, which can be projected onto the external advisor. Thus, “blended” approaches combining digital and personal coaching may be advised in this field of intervention too, which also helps to deal with inevitable issues like fluctuation in staff and restructuring of teams—and is certainly beneficial for integrating the digital solutions into daily routines as well as company structures and strategies. This might also lead to increased adoption and as such to larger sets of data where machine learning can fulfill its potentials. Thus, further research is being conducted on the users' acceptance and adoption of such a technological solution to an inherently social process like team development. Acceptance must also encompass the use of the generated data to personalize the algorithms underlying the wecoach, a potentially powerful feature, but simultaneously also a perceived threat to privacy, despite the guarantee of anonymity and use of quantitative data only. Finally, for researchers in this field, it is vital to understand the challenges of interacting with IT partners and being realistic about the costs to ensure and sustain an attractive and reliable IT system.

## Data Availability Statement

The datasets generated for this study will not be made publicly available as no evaluation results have been presented.

## Author Contributions

All authors listed have made a substantial, direct and intellectual contribution to the work, and approved it for publication.

## Conflict of Interest

GB and GJ are board members of the University Spin-Off that distributes the wecoach. The remaining author declares that the research was conducted in the absence of any commercial or financial relationships that could be construed as a potential conflict of interest.
